# Possibility of new shielding device for upper gastrointestinal endoscopy

**DOI:** 10.1055/a-1523-8959

**Published:** 2021-09-16

**Authors:** Daisuke Kikuchi, Daiki Ariyoshi, Yugo Suzuki, Yorinari Ochiai, Hiroyuki Odagiri, Junnosuke Hayasaka, Masami Tanaka, Tetsuya Morishima, Keita Kimura, Hiroshi Ezawa, Risa Iwamoto, Yoshinori Matsuwaki, Shu Hoteya

**Affiliations:** 1 Department of Gastroenterology, Toranomon Hospital, Tokyo Japan; 2 Olympus Medical Systems Corporation, Tokyo, Japan; 3 Olympus Corporation, Tokyo, Japan; 4 Matsuwaki Clinic Shinagawa, Tokyo, Japan

## Abstract

**Background and study aims** Infection control is essential when performing endoscopic procedures, especially during the COVID-19 pandemic. Therefore, we have developed a new shielding device called STEP for infection control in upper gastrointestinal endoscopy.

**Patients and methods** STEP consists of a mask worn by the patient and a drape that is connected to the mask and covers the endoscope. A suction tube attached to the mask prevents aerosols from spreading. The endoscopist operates the endoscope through the drape. Three endoscopists performed a total of 18 examinations using an upper endoscopy training model with and without STEP. Endoscopic images were evaluated by three other endoscopists, using a visual analog scale. We also simulated contact, droplet, and aerosol infection and evaluated the utility of STEP.

**Results** All examinations were conducted without a problem. Mean procedure time was 126.3 ± 11.6 seconds with STEP and 122.3 ± 10.0 seconds without STEP. The mean visual analog score was 90.7 ± 10.1 with STEP and 90.4 ± 10.0 without STEP. In the contact model, adherence of simulated contaminants was 4.9 ± 1.4 % without STEP and 0 % with STEP. In the droplet model, the number of simulated contaminants attached to the paper was 338 273 ± 90 735 pixels without STEP and 0 with STEP. In the aerosol model, the total number of particles was 346 837 ± 9485 without STEP and was significantly reduced to 222 ± 174 with STEP.

**Conclusions** No effect on examination time or endoscopic image quality was observed when using STEP in upper gastrointestinal endoscopy. Using STEP reduced the diffusion of simulated contaminants in all three infection models.

## Introduction

Many cases of infection and death have been reported due to the coronavirus disease 2019 (COVID-19) pandemic. As of January 2021, more than 85 million cases of infection have been confirmed and the number of deaths has surpassed 1.8 million worldwide.

COVID-19 can develop in individuals infected by severe acute respiratory syndrome coronavirus 2 (SARS-CoV2), which is an RNA virus that belongs to the broader family of coronaviruses. The viruses that caused severe acute respiratory syndrome (SARS) and Middle East Respiratory Syndrome (MERS) were also coronaviruses 1 . SARS-CoV2 is thought to be transmitted mainly by contact infection and droplet infection, but the precise mechanisms are unknown. Recent reports have suggested the possibility of aerosol infection, and thus, more careful infection control measures are considered necessary 2 3 4 . Wearing masks and disinfecting the hands are effective for preventing infection, and medical institutions are also implementing various infection control measures. There is currently no established treatment, but the effectiveness of various antiviral drugs has been reported 5 6 . Vaccine development is being carried out all over the world, and further development is expected in the future 7 8 9 10 11 12 .

Endoscopic procedures carry a risk of infection because there is a risk of exposure to the patient’s respiratory secretions and digestive juices. For this reason, many academic societies have proposed infection control measures for endoscopic procedures 13 14 15 16 17 . When performing endoscopy, it is necessary for endoscopists to wear adequate personal protective equipment (PPE), disinfect their hands, and clean the endoscopy room. In addition to these steps, new steps for infection control that shield patients and prevent the spread of contaminants are considered necessary.

Many cities around the world have been placed on lockdown to slow the spread of the COVID-19 pandemic, which has had serious economic consequences. The pandemic has also affected endoscopic practice, making it difficult to perform conventional endoscopic examinations 18 19 . In response, we are developing the new “Shielding Device for Endoscopic Procedures” as part of the Save the Endoscopy Project (STEP by STEP). Here, we report the current status of the project and report a preliminary evaluation of the feasibility and utility of STEP.

## Patients and methods

### Device description

**Fig. 1:**
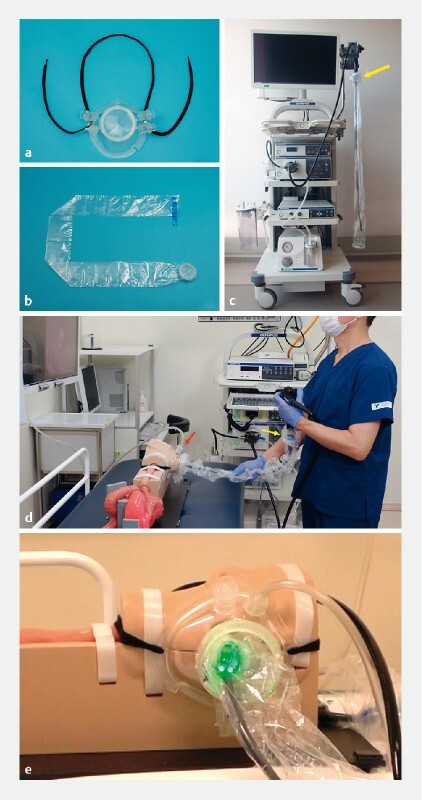
STEP (“Shielding Device for the Endoscopic Procedures”). **a** Mask part, **b** drape part, and **c** endoscope before the examination. The drape was taped to the base of the endoscope (yellow arrow). **d** Simulated endoscopy using STEP. A suction tube was connected to the mask (red arrow) and aerosols generated from the patient were continuously sucked. **e** Simulated endoscopy using STEP. The mask part covered the patient’s nose and mouth.

We are developing STEP in collaboration with Olympus Medical Systems Co., Ltd. and an otolaryngologist. STEP consists of a mask ( Fig. 1a ) worn by the patient and a tubular drape ( Fig. 1b ) that covers the endoscope. On the side of the mask, there is a small hole for air inflow and a suction port for suction of aerosols generated by the patient. The drape connects to a hole at the tip of the mask and the endoscope is inserted into the drape. There is also a valve-like structure in the hole that prevents droplets from escaping when the patient coughs.

### How to use

The use of STEP is shown in Fig. 1c , Fig. 1d , Fig. 1e . First, the endoscope is inserted into the tubular drape, which is then taped to the base of the endoscope. After pharyngeal anesthesia, the mask is placed on the patient’s face and the patient is positioned in the left lateral position as usual. The suction tube is attached to the suction port. Next, the tip of the tubular drape is connected to the hole in the mask. The endoscopist operates the endoscope through the vinyl tubular drape. Because the endoscope is located inside the tubular drape and mask, the endoscopist does not come into direct contact with the patient’s saliva or digestive juices. When the endoscopic procedure is completed, the endoscope is stored inside the tubular drape and the tubular drape is detached from the mask.

Given that the hole in the mask has a valve-like structure, it is difficult for droplets inside the mask to escape. The contaminated endoscope remains in the tubular drape while it is transported to the washing room.

### Experiment

In this study, we conducted experiments to investigate the feasibility and utility of STEP in the upper gastrointestinal endoscopy.

For the feasibility study, we used an EVIS Lucera Elite Gastrointestinal Videoscope (GIF-H290Z; Olympus Medical Systems Corp., Tokyo, Japan) and an upper gastrointestinal endoscopy training model (LM-103; Koken Co., Ltd., Tokyo, Japan). Three endoscopists performed a total of 18 endoscopic examinations on the training model, alternating between using and not using STEP. The endoscopic examination was performed on the pharynx, the upper middle and lower esophagus, and the stomach. The stomach was observed by the screening method proposed by Yao et al. 20 . Then, the duodenal bulb, descending portion, and Vater’s papilla were observed. The procedure time from insertion to removal was compared between the groups using and not using STEP. In addition, three endoscopists who were blinded to the study evaluated endoscopic images of the pharynx/esophagus, the body of the stomach in the retroflex position, the body of the stomach in the straight position, the antrum of the stomach, and the duodenum. The images were evaluated using a visual analog scale (VAS), with scores ranging from 0 (“not observed at all”) to 100 (“completely observed”).

For the utility study, we created models simulating contact, droplet, and aerosol infection.

**Fig. 2:**
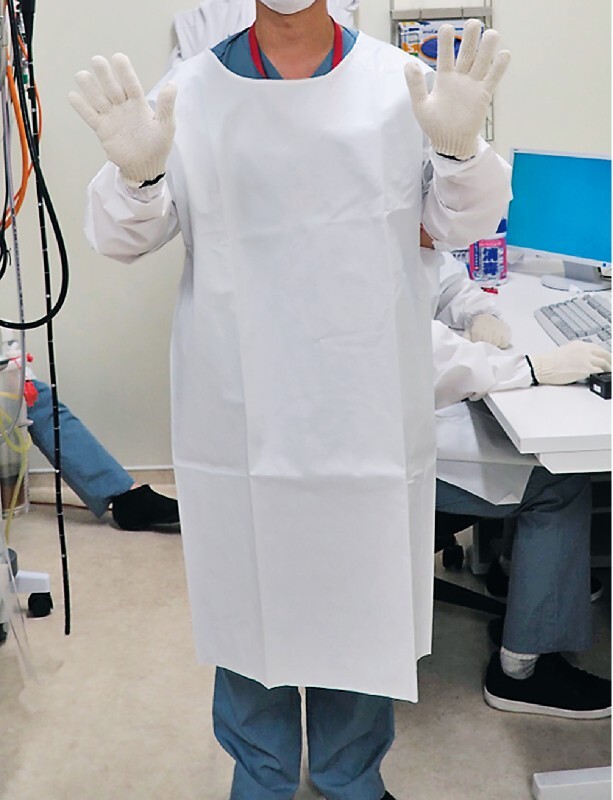
Simulated contact infection model. After spraying indigo carmine pigment into the training model, the endoscopist put on a white glove and performed an endoscopic screening examination. The proportion of the area of the white glove to which the indigo carmine pigment was attached was measured.

In the simulated contact infection model, 20 mL of indigo carmine pigment was sprayed throughout the stomach of the training model. Then, the three endoscopists, who were wearing white gloves, performed the above-mentioned examination ( Fig. 2 ). The ratio of the area of the pigment attached to the white gloves (the area of the attached pigment / the total area of the glove × 100) was measured using image J.

**Fig. 3:**
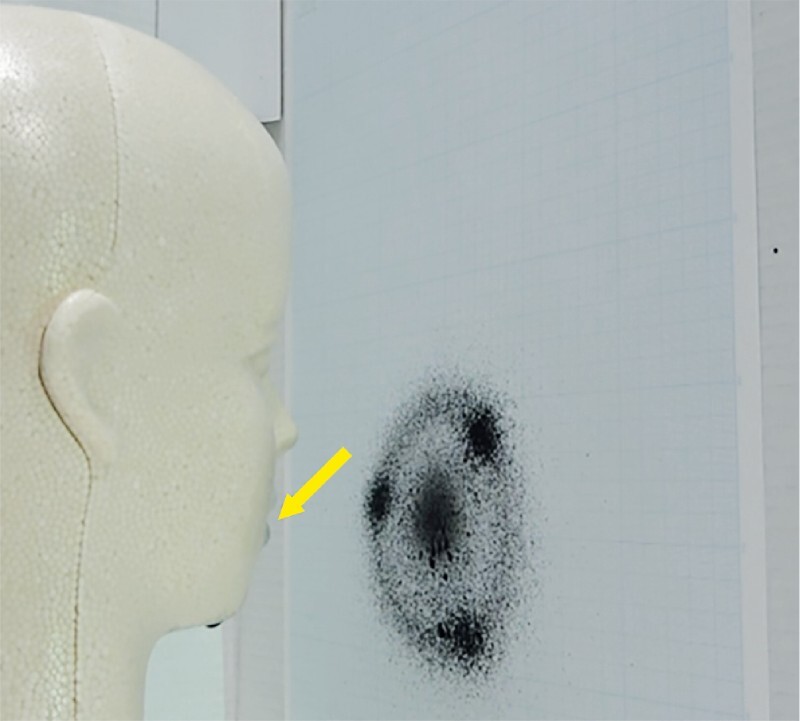
Simulated droplet infection model. A sprayer was installed in the mouth of the head model (yellow arrow) and ink was sprayed. The paper onto which the ink was sprayed was imaged, and the number of pixels that became black after binarization was measured.

In the simulated droplet infection model ( Fig. 3 ), a sheet of paper was hung on a wall 10 cm in front of a sprayer installed in the mouth of a polystyrene foam head model, and 0.2 mL of India ink was sprayed 21 . The paper onto which the India ink was sprayed was then imaged and binarized to divide each pixel into black and white. The experiment was performed three times under the same conditions and the average number of black pixels was compared between trials using and not using STEP.

**Fig. 4:**
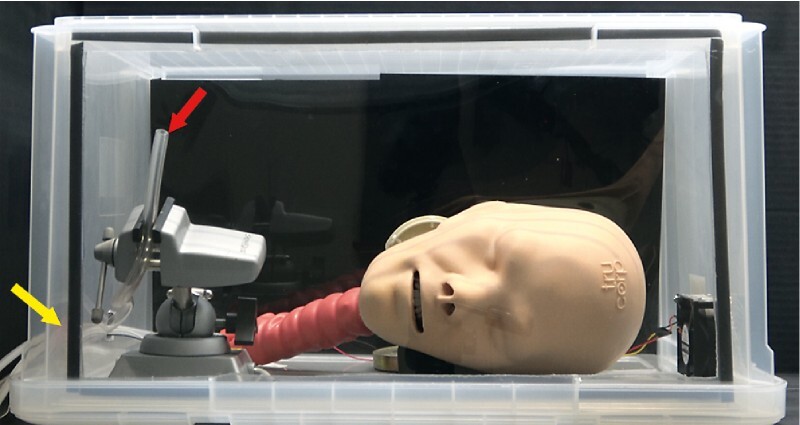
Simulated aerosol infection model. The airway management training model was placed inside the plastic case and the vaporized liquid from an e-cigarette was sprayed through a tube from outside the plastic case (yellow arrow). The number of aerosol particles diffused into the plastic case was measured with a particle counter (red arrow).

In the simulated aerosol infection model 22 23 , an airway management training model (AirSim Advance Bronchi X; TruCorp., Lurgan, UK) and an electronic cigarette (FLEVO; GIEX, Tokyo, Japan) were used ( Fig. 4 ). The airway management training model was placed inside a plastic case to create a stable environment. The vaporized liquid from the electronic cigarette was sprayed from outside the plastic case as a simulated aerosol for 0.9 seconds and then injected into the respiratory tract through a tube. Using a particle counter (MET ONE HHPC 6 + ; Beckman Coulter, Brea, California, United States), the total number of particles 0.3 to 10 μm in size was measured for 120 seconds. The experiment was performed three times under the same conditions and the average total number of particles was compared between trials using and not using STEP. In the trials using STEP, suction was performed from the suction port of the mask with a pressure of −40 kPa and a flow rate of 40 L/min.

### Statistical analysis

Data were analyzed using the unpaired t-test, Chi-squared test, Fisher’s test, or Mann-Whitney U-test as appropriate. P < 0.05 was considered significant. All statistical analyses were performed using SPSS version 20 (SPSS IBM statistics).

## Results

A total of 18 endoscopic procedures were performed using STEP without problem. The procedure time was 126.3 ± 11.6 seconds with STEP and 122.3 ± 10.0 seconds without STEP; no significant difference was observed between the two groups. The VAS scores for the evaluation of endoscopic images taken in the STEP group were as follows: pharynx/esophagus, 91.7 ± 9.0; body of the stomach in the retroflex position, 91.2 ± 9.2; body of the stomach in the straight position, 88.5 ± 12.3; antrum of the stomach, 91.0 ± 9.5; and duodenum, 91.0 ± 11.3. The VAS scores for the non-STEP group were as follows: pharynx/esophagus, 90.9 ± 9.3; body of the stomach in the retroflex position, 91.1 ± 8.8; body of the stomach in the straight position, 87.7 ± 13.7; antrum of the stomach, 90.9 ± 9.0; and duodenum, 91.7 ± 10.4. The results were equivalent for the STEP and non-STEP groups ( Table 1 ).

**Table 1:** Results of the feasibility study

	STEP group (n = 9)	Non-STEP group (n = 9)	*P* value
Procedure time, s ± SD	126.3 ± 11.6	122.3 ± 10.0	N.S.
VAS score			
All images	90.7 ± 10.1	90.4 ± 10.0	N.S.
Pharynx/esophagus	91.7 ± 9.0	90.9 ± 9.3	N.S.
Body of stomach in retroflex position	91.2 ± 9.2	91.1 ± 8.8	N.S.
Body of stomach in straight position	88.5 ± 12.3	87.7 ± 13.7	N.S.
Antrum of stomach	91.0 ± 9.5	90.9 ± 9.0	N.S.
Duodenum	91.0 ± 11.3	91.7 ± 10.4	N.S

Data are shown as the mean ± standard deviation.

STEP, ; N.S., not significant; VAS, visual analog scale.

In the contact infection model, the proportion of the area of the white gloves onto which the indigo carmine pigment adhered was 4.9 ± 1.4 % in the non-STEP group and 0 % in the STEP group.

In the droplet infection model, the number of pixels that became black after binarization was 351 418 ± 90 735 in the non-STEP group and 0 in the STEP group.

In the aerosol infection model, the average total number of particles in the plastic case was 346 837 ± 9485 in the non-STEP group and was significantly reduced to 222 ± 174 in the STEP group ( Table 2 ).

**Table 2:** Results of the utility study

	STEP group (n = 3)	Non-STEP group (n = 3)	*P* value
Contact infection model Proportion of area of the white glove to which the indigo carmine pigment adhered (% ± SD)	0	4.9 ± 1.4	*P* < 0.05
Droplet infection model Number of pixels that became black after binarization (pixels ± SD)	0	351 418 ± 90 735	*P* < 0.05
Aerosol infection model Total number of particles in the plastic case (number ± SD)	222 ± 174	346 837 ± 9485	*P* < 0.05

Data are shown as the mean ± standard deviation.

SD, standard deviation.

## Discussion

Endoscopic procedures, especially upper gastrointestinal endoscopy, carry a risk of contact, droplet, and aerosol infection, and thus appropriate infection control measures are required. Current infection control consists of the following three steps: before the procedure, the endoscopy staff disinfects their hands; during the procedure, the endoscopy staff wears PPE; after the procedure, the endoscopy staff cleans the endoscope and endoscopy room and disinfects their hands 24 . However, once contaminants are diffused by coughing or vomiting, it is difficult to completely clean and disinfect objects they come into contact with. Therefore, these three steps are considered to be insufficient as infection control measures. It is necessary to shield the patient in order to prevent as much as possible the spread of contaminants to the surrounding environment. Accordingly, we have developed the STEP shielding device for endoscopic procedures.

Several methods for shielding patients during endoscopy have previously been reported 25 26 27 28 29 30 31 , including a transparent plastic box and a vinyl sheet, both of which cover the patient. However, these devices need to be cleaned and disinfected if they are reused. Therefore, disposable items are desirable and we developed a solution consisting of a disposable mask and drape. In addition, it was considered that the operability of the endoscope may deteriorate when the patient is covered with a shield during the procedure. We were similarly concerned that STEP might also affect the operability of the endoscope. However, although the upper gastrointestinal endoscopy took several seconds longer when using STEP, no significant difference was observed between the STEP and non-STEP groups. In addition, the evaluation of endoscopic images produced nearly identical results between the STEP and non-STEP groups. It was considered that the effect of STEP on the operability of the upper endoscopy was minimal.

The primary purpose of shielding is to prevent contact with droplets from the patient’s mouth and nose. However, saliva and digestive juices attached to the endoscope can also be a source of infection. STEP’s tubular drape is attached to the endoscope and prevents direct contact with saliva and digestive juices. In addition, given that the endoscope can remain inside the tubular drape while being carried to the washing room, it is possible to prevent contamination of the surrounding environment and medical staff after the examination. In other words, STEP can play an important role not only during but also after the endoscopic procedure. In this experiment, there was zero contact with simulated contaminants in the STEP group in both the contact infection and droplet infection models. Endoscopic procedures are also considered to pose a risk of aerosol infection. COVID-19 can be spread by aerosol infection as well as contact and droplet infection. To prevent aerosols from spreading, a suction port was added to the mask. Continuous suction during the procedure significantly reduced the amount of simulated aerosols released into the surrounding environment.

This study has several limitations. First, it is unclear how many droplets and aerosols are generated during actual endoscopic procedures and how infectious they are. In this experiment, an electronic cigarette was used as a simulated aerosol, but there are no data on aerosol generation in clinical practice. In the future, we plan to measure droplets and aerosols the clinical setting and investigate the extent to which they can be contained by STEP. Second, if the patient’s oxygen saturation level drops, it is necessary to remove the mask and apply a nasal cannula for supplemental oxygen. At this time, the patient might cough or sneeze and spread droplets and aerosols. Therefore, nasal cannulas should be applied before the start of the procedure when administering sedative agents. In addition, it is considered important to monitor the oxygen saturation level during an endoscopic procedure performed with STEP. It is expected that the patient might feel some discomfort from wearing the mask during the procedure. Moreover, saliva and vomiting may also collect in the mask, which an increase the patient’s discomfort and affect the breathing. Therefore, before introducing STEP into clinical practice, we plan to prospectively evaluate patient discomfort and oxygen saturation level. Third, STEP’s tubular cover may affect the operability of the endoscope slightly. In this experiment, no significant difference was observed between screening endoscopy performed with and without STEP, but it is necessary to investigate whether STEP affects operability when used in therapeutic endoscopy. Finally, major issues are cost and the question of whether to use STEP for all examinations and treatments or for only patients at high risk of infection. It is also important to evaluate the time required to prepare and dispose of the STEP when introducing it into clinical practice. Therefore, it will be necessary to evaluate the advantages and disadvantages of STEP, including its cost and time.

## Conclusions

In this study, we developed STEP in collaboration with Olympus Medical Systems Co., Ltd. and an otolaryngologist. When using STEP, the operability of the upper gastrointestinal endoscope and the quality of the obtained endoscopic images were equivalent to the conventional method without STEP. In addition, the use of STEP made it possible to reduce the spread of contaminants in simulated models of contact, droplet, and aerosol infection, suggesting that STEP may be useful for infection control in upper gastrointestinal endoscopy.
